# Sustained effect of LACTIN-V (*Lactobacillus crispatus* CTV-05) on genital immunology following standard bacterial vaginosis treatment: results from a randomised, placebo-controlled trial

**DOI:** 10.1016/S2666-5247(22)00043-X

**Published:** 2022-04-21

**Authors:** Eric Armstrong, Anke Hemmerling, Steve Miller, Kerianne E Burke, Sara J Newmann, Sheldon R Morris, Hilary Reno, Sanja Huibner, Maria Kulikova, Nico Nagelkerke, Bryan Coburn, Craig R Cohen, Rupert Kaul

**Affiliations:** Department of Medicine (E Armstrong BSc, S Huibner BSc, B Coburn PhD, Prof R Kaul MD), Department of Laboratory Medicine and Pathobiology (B Coburn), and Department of Immunology, University of Toronto, Toronto, ON, Canada (B Coburn, Prof R Kaul); Department of Obstetrics, Gynecology, and Reproductive Sciences (A Hemmerling MD, Prof S J Newmann MD, Prof C R Cohen MD) and Department of Laboratory Medicine (Prof S Miller MD), University of California, San Francisco, CA, USA; Department of Family Medicine and Public Health, University of California, San Diego, CA, USA (S R Morris MD); Ruth M Rothstein CORE Centre, John H Stroger Jr Hospital of Cook County, Chicago, IL, USA (K E Burke MPH); Department of Medicine, Washington University, St Louis, MO, USA (H Reno MD); Toronto General Hospital Research Institute (M Kulikova DPT, B Coburn) and Department of Medicine (B Coburn, Prof R Kaul), University Health Network, Toronto, ON, Canada; Centre for Global Health Research, St Michael’s Hospital, Unity Health Toronto, Toronto, ON, Canada (Prof N Nagelkerke PhD)

## Abstract

**Background:**

Bacterial vaginosis might increase HIV risk by eliciting genital inflammation and epithelial barrier disruption, whereas vaginal *Lactobacillus crispatus* is associated with immune quiescence and HIV protection. We investigated the effect of a live biotherapeutic containing *L crispatus* CTV-05 (LACTIN-V) on genital immunology and key vaginal bacteria.

**Methods:**

This substudy included women aged 18–45 years who participated in the randomised, placebo-controlled, phase 2b trial of LACTIN-V to reduce bacterial vaginosis recurrence, conducted at four universities and hospitals in the USA. Women with negative results for sexually transmitted infection, pregnancy, and urinary tract infection were provided a 5-day course of vaginal metronidazole 0·75% gel. Those who met at least three of four clinical Amsel criteria for bacterial vaginosis and had a Nugent score of 4–10 from Gram staining were eligible. Participants in the LACTIN-V trial were randomly assigned (2:1) to receive either LACTIN-V or placebo, applied vaginally once per day for 5 days during the first week and then twice per week for 10 more weeks. Follow-up visits occurred 4, 8, 12, and 24 weeks after enrolment. Soluble immune factors and the absolute abundance of bacterial taxa were assayed by mutliplex ELISA and quantitative PCR. The primary outcomes were vaginal levels of IL-1α and soluble E-cadherin at 24 weeks (ie, 13 weeks after treatment cessation).

**Findings:**

Between Feb 21, 2020 and March 18, 2021, we characterised genital immune parameters and the vaginal microbiota in a subset of 66 highly adherent participants who were randomly selected, with no exclusion criteria, from those who had attended all study follow-up visits (n=166) in the larger LACTIN-V clinical trial (n=288). 32 (48%) participants received LACTIN-V and 34 (52%) received placebo. LACTIN-V treatment was significantly associated with lower concentrations of the proinflammatory cytokine IL-1α (β coefficient 0·310, SE 0·149; p=0·042) and soluble E-cadherin (0·429, 0·199; p=0·035), a biomarker of epithelial barrier disruption.

**Interpretation:**

Vaginal administration of LACTIN-V following standard bacterial vaginosis therapy resulted in a sustained reduction in genital inflammation and a biomarker of epithelial integrity. The potential of LACTIN-V to reduce HIV susceptibility merits further investigation.

**Funding:**

Canadian Institutes of Health Research and the National Institutes of Health National Institute of Allergy and Infectious Diseases.

## Introduction

Bacterial vaginosis has been linked to adverse reproductive health outcomes among women and to increased risk of HIV acquisition.^1,2^ Bacterial vaginosis is characterised by diverse anaerobic bacteria^3,4^ and might elevate HIV risk by eliciting genital inflammation, which not only recruits HIV-susceptible CD4^+^ T cells to mucosal tissues but also causes epithelial barrier disruption with the cleavage of E-cadherin, a key component of epithelial cell-to-cell junctions,^5,6^ thereby increasing viral access to these target cells.^7–9^ Although standard antibiotic therapy for bacterial vaginosis rapidly reduces vaginal prototypic inflammatory cytokine IL-1α,^10^ treatment has been shown to increase vaginal concentrations of some chemoattractant immune factors linked to HIV risk, such as IFN-γ-induced protein (IP)-10,^9,11^ and so the immununological benefits of treatment remain uncertain.

In the absence of bacterial vaginosis, one of several species of *Lactobacillus* is typically predominant in the vaginal microbiome and has been linked to protection against HIV acquisition.^3,12^ For example, *Lactobacillus crispatus* predominance in the female genital tract has been linked to protection against HIV acquisition,^7,13^ most likely due to direct anti-inflammatory effects^6,14^ or the competitive exclusion of proinflammatory bacterial vaginosis-associated bacteria, or both.^15^ The standard of care for clinical bacterial vaginosis treatment involves oral or topical antibiotics targeting bacterial vaginosis-associated anaerobes, but recurrence rates are high^16^ and treatment does not always result in predominance of the protective *L crispatus* species.^11^ Although studies have investigated *Lactobacillus* spp-based probiotics or live biotherapeutics as alternative treatment strategies for bacterial vaginosis, none have linked probiotic use to reduced genital inflammation, which might be because of the use of probiotics containing *Lactobacillus* species that are not commonly predominant in the female genital tract.^17–19^ A phase 2 trial investigating the vaginal administration of a live biotherapeutic containing the *L crispatus* strain CTV-05 (LACTIN-V) following standard antibiotic treatment for bacterial vaginosis found that participants receiving LACTIN-V had lower rates of bacterial vaginosis recurrence than those receiving placebo, and these benefits were sustained for at least 3 months after treatment cessation.^20^

We investigated whether LACTIN-V administration has a sustained effect on genital immunology, and the associations between immune alterations and vaginal microbiota.

## Methods

### Study design and participants

This substudy included women aged 18–45 years who participated in the randomised, placebo-controlled, phase 2b trial^20^ of LACTIN-V to reduce bacterial vaginosis recurrence, conducted at four universities and hospitals in the USA. The trial evaluated the ability of LACTIN-V to prevent bacterial vaginosis recurrence following standard antibiotic treatment. At entry screening, the participants’ medical history was obtained, and physical and pelvic examinations were performed. Women with negative results for sexually transmitted infection (STI), pregnancy, and urinary tract infection (appendix p 1) were provided a 5-day course of vaginal metronidazole 0·75% gel. Within 48 h of completing treatment, participants returned to the clinic and those who met at least three of four clinical Amsel criteria for bacterial vaginosis and had a Nugent score of 4–10 from Gram staining (done at screening before antibiotic treatment) were eligible for the trial. The LACTIN-V trial (NCT02766023) obtained written consent from all participants before enrolment. Study protocols for the larger trial were approved by the institutional review boards (IRBs) at University of California, San Francisco General Hospital (San Francisco, CA, USA; IRB 15-18143), Stroger Hospital of Cook County (Chicago, IL, USA), University of California San Diego Antiviral Research Center (San Diego, CA, USA; IRB: 160023X), and Washington University Infectious Disease Clinical Research Unit (St Louis, MO, USA). Immunology and microbiology performed at the University of Toronto (Toronto, ON, Canada) were reviewed and approved by the University of Toronto HIV Research Ethics Board (protocol #36947).

### Procedures

Participants in the LACTIN-V trial were randomly assigned (2:1) to receive either LACTIN-V or placebo, which were applied vaginally once per day for 5 days during the first week and then twice per week for 10 more weeks. Participants returned for follow-up visits at 4, 8, 12, and 24 weeks after enrolment. Vaginal swabs were collected at all visits (appendix p 1).

For the soluble immune factor measurement, cervico-vaginal fluid obtained from vaginal swabs was thawed and centrifuged at 4500 rpm for 30 min. Supernatant was removed for immune factor analysis and the bacterial pellet was left intact for quantitative PCR (qPCR) analyses. The soluble immune factors IL-1α, IFN-α2A, IL-17A, IL-6, IFN-γ, IP-10, IL-8, macrophage inflammatory protein (MIP)-1β, MIP-3α, monokine induced by IFN-γ (MIG), soluble E-cadherin, and matrix metalloproteinase (MMP)-9 were measured in duplicate with mutliplex ELISA on the Meso Scale Discovery platform (Meso Scale Diagnostics, Rockville, MD, USA).^21^

At the University of Toronto, DNA was extracted from 175 μL of bacterial pellet using the Qiagen DNEasy PowerSoil Kit (Qiagen, Valencia, CA, USA). Targeted qPCR was used to estimate the absolute abundance of key bacterial species. At the University of California, San Francisco, DNA was extracted from 200 μL of vaginal sample using the EZ1 DNA Tissue Kit (Qiagen, Valencia, CA, USA) and eluted into 50 μL volume. The absolute abundance of *L crispatus* and *L crispatus* CTV-05 were estimated with qPCR. Details on microbiome analyses are provided in the appendix (p 1).

### Outcomes

The primary outcomes were the vaginal IL-1α and soluble E-cadherin concentrations at 24 weeks (ie, 13 weeks after treatment cessation). Exploratory outcomes included vaginal concentrations of soluble immune factors IP-10, IL-6, IL-8, MIP-1b, MIP-3a, MIG, and MMP-9, and the absolute abundance of key vaginal bacteria taxa including *L crispatus*, *L crispatus* strain CTV-05, *Lactobacillus iners*, *Lactobacillus jensenii*, *Lactobacillus gasseri*, *Gardnerella vaginalis*, *Atopobium vaginae*, *Megasphaera* species, and *Prevotella* species.

### Statistical analysis

After visual inspection of distribution, soluble immune factor concentrations and bacteria copy numbers were normalised using log_10_ transformation. Linear regression was used to evaluate the association between treatment group as an independent variable and both the concentrations of soluble immune factors and abundance of key vaginal bacteria taxa at 24 weeks as dependent variables. Soluble immune factors that were less than the lower limit of detection in more than 50% of participants were dichotomised as detectable and undetectable. Binary logistic regression was used to evaluate the association between treatment group and the detectability of soluble immune factors at 24 weeks. For all regression models that predicted concentrations and the detectability of soluble immune factors or abundance of vaginal bacteria taxa, baseline measurements of the dependent variable were included in the model to control for interindividual variability. To evaluate potential mediating effects of bacterial taxa on the relationship between LACTIN-V and soluble immune factors, we followed the framework by Baron and Kenny^22^ outlined in the appendix (p 1). Mann-Whitney *U* tests were performed to compare the change in soluble immune factors from baseline to 24 weeks between women with vaginal predominance of non-CTV-05 *L crispatus* and those with predominance of *L crispatus* CTV-05. All statistical tests were performed with SPSS (version 270.0.0) or GraphPad Prism (version 9.0.2).

### Role of the funding source

The funder of the study had no role in study design, data collection, data analysis, data interpretation, or writing of the report.

## Results

Between Feb 21, 2020 and March 18, 2021, we characterised genital immune parameters and the vaginal microbiota in a subset of 66 highly adherent participants who were randomly selected, with no exclusion criteria, from those who had attended all study follow-up visits (n=166) in the larger LACTIN-V clinical trial (n=288);^20^ 32 (48%) participants received LACTIN-V and 34 (52%) received placebo.

Baseline samples were missing from three participants who received placebo; immune and microbiota analyses (except for *L crispatus* CTV-05 quantitation, which had been performed earlier) were not performed on these samples. Baseline participant characteristics and sexual behaviours, including recent sex and hormonal contraceptive use did not substantially differ between groups (table 1). All participants were women, with a median age of 33 years (IQR 27–38). As per eligibility criteria, none of the women tested positive for HIV, syphilis, *Neisseria gonorrhoea*, *Chlamydia trachomatis*, or *Trichomonas vaginalis* at baseline. All participants reported the application of at least 75% of LACTIN-V doses, which was confirmed with trypan blue staining of applicators.^20^

**Figure 1:**
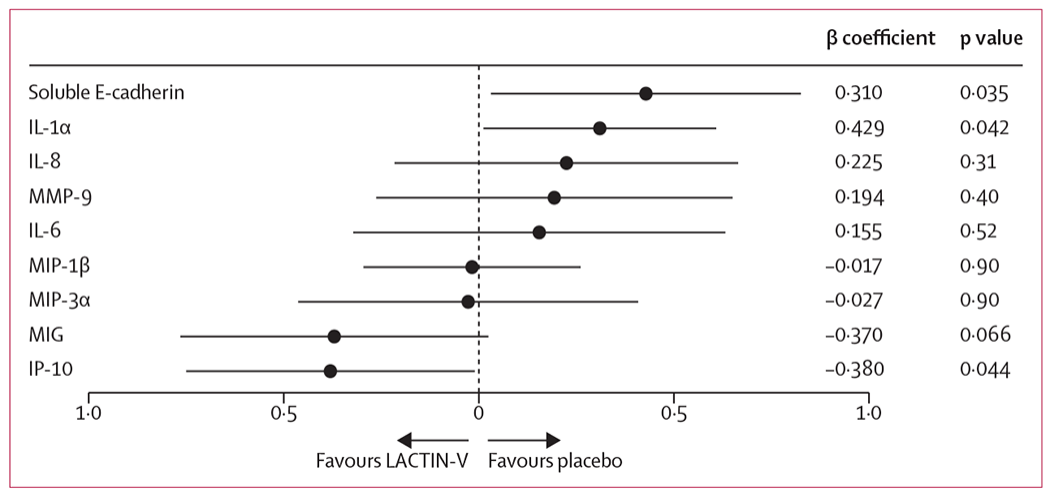
Association between LACTIN-V treatment and vaginal soluble immune factors at 24 weeks Data are β coefficient. Error bars are 95% CIs.

LACTIN-V treatment was significantly associated with lower concentrations of the proinflammatory cytokine IL-1α (β coefficient 0·310, SE 0·149; p=0·042) and soluble E-cadherin (0·429, 0·199; p=0·035), a biomarker of epithelial barrier disruption (figure 1). Among the six additional immune factors that were included as exploratory endpoints, LACTIN-V was associated with elevated concentrations of IP-10 at 24 weeks (−0·380, 0·185; p=0·044; figure 1). IFN-α2a and IL-17A were undetectable in more than 50% of samples, so were dichotomised as detectable or undetectable. Detectability of IFN-α2a or IL-17A was not significantly associated with the treatment group (appendix p 6).

We hypothesised that an altered abundance of specific vaginal bacteria were causing the sustained differences between treatment groups in genital immune parameters. LACTIN-V treatment was associated with a sustained increase in the abundance of *L crispatus* (β −2·085, SE 0·649; p=0·0021) and *L gasseri* 13 weeks after treatment cessation (−0·803, 0·346; p=0·024; table 2). LACTIN-V treatment was also associated with a sustained reduction in the abundance of the bacterial vaginosis-associated genus *Prevotella* spp (1·033, 0·509; p=0·047) and borderline significance of reduced *Megasphaera* spp abundance (1·452, 0·731, p=0·052). *L crispatus* was more likely to be detected 13 weeks after product cessation among participants who received LACTIN-V (68·8% *vs* placebo 31·2%; p=0·0002) and the *L crispatus* strain CTV-05 was also present in those who received LACTIN-V (53·1% *vs* 2·9%; p<0·0001; appendix p 2). Among participants with previous LACTIN-V treatment and a high abundance of *L crispatus* (>1×10^6^ copies per mL) at 24 weeks, the *L crispatus* strains present were predominantly non-CTV-05 in seven (41%) of 17 participants and predominantly CTV-05 in ten (59%; appendix p 4).

LACTIN-V administration was associated with the concentrations of soluble immune factors and abundances of multiple bacterial taxa. To explore potential causal pathways underpinning these relationships, we assessed the mediating effects of key bacterial taxa on the relationship between LACTIN-V treatment and concentrations of IL-1α, soluble E-cadherin, and IP-10 at 24 weeks. Change in IL-1α was positively associated with change in the abundance of *Prevotella* spp (β 0·179, SE 0·039; p<0·0001) and *Megasphaera* spp (0·085, 0·026; p=0·0016; table 3). Change in soluble E-cadherin was negatively associated with change in *L crispatus* (−0·128, 0·036; p=0·0007) and positively associated with change in *Prevotella* spp (0·338, 0·038; p<0·0001) and *Megasphaera* spp (0·157, 0·029; p<0·0001). Change in IP-10 was positively associated with change in *L crispatus* (0·108, 0·035; p=0·0029) and negatively associated with change in *Prevotella* spp (−0·161, 0·050, p=0·0022) and *Megasphaera* spp (−0·135, 0·029; p<0·0001).

Next, we assessed whether genital immune changes associated with LACTIN-V were mediated through treatment-elicited alterations in vaginal bacteria. When change in IL-1α was the dependent variable, there was a significant association with change in *Prevotella* spp (β 0·167, SE 0·039; p<0·0001; table 3) and *Megasphaera* spp (0·075, 0·026, p=0·0058), but not with LACTIN-V treatment. Using change in soluble E-cadherin as the dependent variable, there were associations with change in *L crispatus* (−0·114, 0·038, p=0·0037), *Prevotella* spp (0·327, 0·038; p<0·0001), and *Megasphaera* spp (0·148, 0·030; p<0·0001), but not with LACTIN-V treatment. Similarly, change in IP-10 was not significantly associated with the treatment group, but was positively associated with change in *L crispatus* (0·096, 0·037; p=0·011) and negatively associated with change in *Prevotella* spp (−0·148, 0·051, p=0·0051) and *Megasphaera* spp (−0·128, 0·029; p<0·0001).

**Figure 2:**
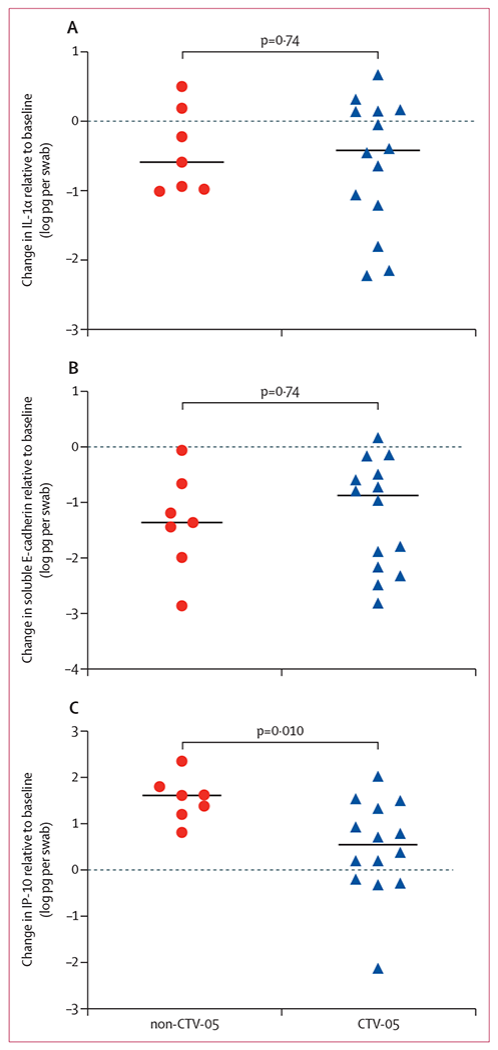
Change in soluble immune factors from baseline to 24 weeks Horizonal line indicates median. For participants with high *Lactobacillus crispatus* abundance (>1 × 10^6^ copies per mL), the change in vaginal IL-1α (A), soluble E-cadherin (B), and IP-10 (C) was compared between groups with sustained predominance by the *L crispatus* non-CTV-05 strain versus the LACTIN-V *L crispatus* CTV-05 strain.

Finally, we investigated whether there was a differential immune effect of post-treatment vaginal colonisation with the CTV-05 strain of *L crispatus*. Of 21 selected participants with high vaginal abundance of *L crispatus* at 24 weeks, 14 (67%) showed CTV-05 strain predominance and seven (33%) showed non-CTV-05 strain predominance. Although predominance by CTV-05 or non-CTV-05 strains of *L crispatus* was non-significantly associated with similar reductions in IL-1α and soluble E-cadherin (p=0·74 for both), increases in vaginal IP-10 concentrations were significantly greater among participants with predominance of non-CTV-05 strains compared with predominance of the CTV-05 strain after LACTIN-V treatment (p=0·010; figure 2).

## Discussion

Vaginal application of the novel *L crispatus*-based live biotherapeutic LACTIN-V after bacterial vaginosis treatment with topical metronidazole reduces recurrence, with effects sustained for 3 months after the last dose of LACTIN-V.^20^ In this study, we show that LACTIN-V treatment was associated with similarly sustained reductions in genital mucosal inflammation and a biomarker of epithelial barrier disruption. Echoing findings from a study examining the immune effect of standard bacterial vaginosis therapy,^11^ secondary analysis showed an increase in vaginal concentrations of IP-10 (a chemoattractant chemokine linked to increased risk of HIV acquisition in women) after successful bacterial vaginosis treatment.^9,23^ These sustained genital immune effects were primarily mediated by a reduced abundance of bacterial vaginosis-associated taxa, particularly *Prevotella* spp and *Megasphaera* spp, and to a lesser extent by an elevated abundance of *L crispatus*. Increases in vaginal IP-10 were specifically linked to abundance of non-CTV-05 *L crispatus* strains, rather than to the therapeutic CTV-05 strain.

Rapid bacterial vaginosis recurrence following standard antibiotic treatment has generated substantial interest in alternative treatment strategies and antibiotic adjuncts.^16^ Among these strategies is the use of non-*crispatus Lactobacillus* probiotics or live biotherapeutics, either alone or in combination with standard antibiotics. However, not all probiotics have been shown to reduce rates of bacterial vaginosis recurrence,^24^ and the three studies that have evaluated genital immunology showed little or no effect of *Lactobacillus* probiotics,^17,18^ or an increase in proinflammatory cytokines following probiotic use.^19^ The present study is the first to show that application of an *L crispatus*-based live biotherapeutic after standard topical metronidazole therapy reduces concentrations of a proinflammatory genital cytokine and a marker of epithelial disruption. An important distinction between the present study and previous studies was the use of a live biotherapeutic comprised entirely of *L crispatus*, a species of *Lactobacillus* known to naturally predominate in the vagina,^3^ which has been associated with genital immune benefits and HIV protection among women.^7,14,25^

In a longitudinal cohort study,^7^ vaginal predominance by *L crispatus*, but not *L iners*, was associated with reduced risk of HIV acquisition compared with a bacterial vaginosis-type microbiota. Whether the HIV-protective effects of *L crispatus* are due to direct anti-inflammatory effects, the exclusion of bacterial vaginosis-associated bacteria, or other mechanisms is unclear. Vaginal predominance by *L crispatus* is associated with reduced vaginal concentrations of proinflammatory cytokines, including IL-1α,^14^ and our group has linked an increased *L crispatus* proportional abundance to reduced concentrations of genital proinflammatory cytokines and chemokines.^25^ Women with *L crispatus* predominance also exhibit fewer transitions to a bacterial vaginosis-type microbiota than women with *L iners* predominance, suggesting that *L crispatus* can competitively exclude inflammatory bacterial vaginosis-associated bacteria,^26^ mediated partly by the production of lactic acid and antimicrobial metabolites.^27^ In vitro models show that *L crispatus* is a key producer of lactic acid in the female genital tract,^27^ which might directly dampen the production of proinflammatory cytokines.^28,29^ Furthermore, addition of supernatant with *L crispatus* or *L crispatus* alone, or both, to epithelial cells decreased the production of proinflammatory cytokines relative to bacterial vaginosis-associated bacteria,^6,14^ and protected against epithelial barrier disruption in response to proinflammatory stimuli.^6^ Reduced genital inflammation following LACTIN-V could be mediated by the exclusion of bacterial vaginosis-associated bacteria, although our study showed that *L crispatus* might have direct but minor effects on concentrations of soluble E-cadherin and IP-10.

Observational studies have linked bacterial vaginosis with reduced vaginal concentrations of IP-10,^10,25^ suggesting that either the absence of bacterial vaginosis-associated bacteria or presence of *Lactobacillus* spp might elicit IP-10.^10^ Joag and colleagues^11^ showed that successful antibiotic bacterial vaginosis treatment resulting in an *L iners* predominant vaginal microbiome increased vaginal concentrations of several chemokines at 1 month, including IP-10 and MIG. In this study, we provide evidence that the reduced concentrations of soluble E-cadherin and elevated concentrations of IP-10 observed among participants receiving LACTIN-V were mediated partly by an elevated abundance of non-CTV-05 *L crispatus* strains. This finding suggests that *L crispatus* CTV-05 (LACTIN-V) might reduce genital inflammation and enhance epithelial barrier integrity in the same way as non-CTV-05 *L crispatus*, without eliciting an elevation in proinflammatory chemokines. Importantly, CTV-05 was detected in one participant who received placebo, probably because the CTV-05 strain found in LACTIN-V is derived from a naturally occurring vaginal strain of *L crispatus*.

There are several limitations of our study. First, STIs were routinely assessed at screening, but afterwards were only tested if women presented with symptoms. Therefore, we were unable to control for the presence of asymptomatic STIs during the study, which might affect genital immunology at follow-up visits. However, we would expect a low incidence of STIs because all participants were negative for STIs at trial screening, diagnostics were performed in the context of any genital symptoms, and the overall 6-month incidence of STIs is low in sexually active women in the USA.^30^ Second, the stage of the menstrual cycle was not recorded at each visit, except for menstruation. Although no study visits occurred during menstruation, this limitation reduced our ability to control for fluctuations in sex hormones, a potential determinant of genital immunology. Third, targeted qPCR rather than 16S rRNA gene sequencing or metagenomic sequencing was used to show the association of cervicovaginal IP-10 concentrations with non-CTV-05 *L crispatus* strains, limiting our ability to evaluate the vaginal microbiota and identify contributions by other microbes. Fourth, the study population consisted only of US American women, albeit a heterogeneous cohort in terms of ethnicity. Finally, the immunology analyses involved a subset of participants who had attended all trial visits in a larger clinical trial, which had strict eligibility criteria.^20^ Therefore, although we believe that these results reflect a causal relationship between LACTIN-V and genital immunology, they should be viewed as hypothesis generating. To ensure generalisability and definitively show causation, future studies are needed.

Strong links between the vaginal microbiota and HIV risk suggest that promoting vaginal predominance by *L crispatus* might reduce HIV risk. Treatment with LACTIN-V following standard antibiotics has been shown to reduce bacterial vaginosis recurrence compared with placebo, even at 3 months after the last dose.^20^ In this study, we show that LACTIN-V treatment was also associated with sustained reductions in biomarkers of genital inflammation and epithelial barrier damage, through the promotion of increased *L crispatus* abundance and decreased abundance of *Prevotella* species and *Megasphaera* species. Additionally, LACTIN-V was associated with elevated concentrations of the proinflammatory chemokine IP-10, although this finding appeared to be driven by non-CTV-05 *L crispatus* rather than *L crispatus* CTV-05. Future work is needed to determine whether LACTIN-V administration following standard antibiotics can reduce HIV acquisition among women at high risk.

## Supplementary Material

1

## Figures and Tables

**Table 1:** Participant characteristics

	LACTIN-V (n=32)	Placebo (n=34)
**Sociodemographic factors**		

Median age, years	32 (27–36)	34 (27–38)
Ethnicity		
Asian	3 (9%)	2 (6%)
Black or African American	14 (44%)	15 (44%)
Multiracial	0	2 (6%)
White	14 (44%)	12 (35%)
Unknown	1 (3%)	3 (9%)

**Sexual behaviours, intravaginal practices, and contraception**		

Vaginal sex within 30 days of enrolment	24 (75%)	30 (88%)
Vaginal sex between the 24-week visit and the previous visit	21 (66%)	27 (79%)
Douching or intravaginal practices at any time	12 (38%)	8 (24%)
Douching or intravaginal practices between the 24-week visit and the previous visit	3 (9%)	2 (6%)
Hormonal contraception use	8 (25%)	10 (29%)

Data are median (IQR), n (range), or n (%). Data on age, race, and hormonal contraceptive use were collected at baseline, and all the other data were collected at baseline and the 24-week visit.

**Table 2:** Association between the treatment group and absolute abundance of key bacterial taxa at 24 weeks

	β coefficient	SE	p value
*Lactobacillus crispatus* *	−2·085	0·649	0·0021
*Lactobacillus iners*	0·383	0·449	0·397
*Lactobacillus gasseri* *	−0·803	0·346	0·024
*Lactobacillus jensenii*	−0·894	0·557	0·11
*Prevotella* spp*	1·033	0·509	0·047
*Gardnerella vaginalis*	0·996	0·543	0·072
*Megasphaera* spp*	1·452	0·731	0·052
*Atopobium vaginae*	0·633	0·727	0·39

Baseline measurements of bacteria absolute abundance (defined as log_10_ transformed copy numbers) were included in each model to control for interindividual variation.

* Represents discrete models.

**Table 3:** Associations between the change in soluble immune factors and absolute abundance of key vaginal bacteria from baseline to 24 weeks

	Change in IL-1α	p value	Change in soluble E-cadherin	p value	Change in IP-10	p value
**Univariable models**						

Change in Lactobacillus crispatus*	−0·050 (0·031)	0·11	−0·128 (0·036)	0·0007	0·108 (0·035)	0·0029
Change in *Lactobacillus gasseri*	−0·011 (0·059)	0·85	−0·106 (0·074)	0·16	0·129 (0·069)	0·067
Change in *Prevotella* spp*	0·179 (0·039)	<0·0001	0·338 (0·038)	<0·0001	−0·161 (0·050)	0·0022
Change in *Megasphaera* spp	0·085 (0·026)	0·0016	0·157 (0·029)	<0·0001	−0·135 0·029	<0·0001

**Multivariable models**						

Change in Lactobacillus crispatus*	−0·033 (0·032)	0·30	−0·114 (0·038)	0·0037	0·096 (0·037)	0·011
LACTIN-V treatment group*	0·333 (0·204)	0·11	0·271 (0·243)	0·27	−0·235 (0·237)	0·32
Change in *Prevotella* spp	0·167 (0·039)	<0·0001	0·327 (0·038)	<0·0001	−0·148 (0·051)	0·0051
LACTIN-V treatment group	0·254 (0·175)	0·15	0·218 (0·169)	0·20	−0·307 (0·226)	0·18
Change in *Megasphaera* spp*	0·075 (0·026)	0·0058	0·148 (0·030)	<0·0001	−0·128 (0·029)	<0·0001
LACTIN-V treatment group*	0·264 (0·189)	0·17	0·235 (0·215)	0·28	−0·204 (0·213)	0·34

Data are unstandardised β coefficients (SE). Change in log_10_ transformed copy number for all bacterial taxa and log_10_ transformed concentrations of soluble immune factors. Bacterial taxa only included if significantly associated with the treatment group and at least one of the soluble immune factors: IL-1α, E-cadherin, and IP-10.

* Represents discrete models.

## Research in context

### Evidence before this study

We performed a PubMed search using the search terms “(“immun*” OR “inflammat*” OR HIV) AND (“bacterial vaginosis” AND “treat*” AND (probiotic OR “live biotherap*”)) NOT (review[Publication Type])” from database inception to July 16, 2021, which returned 56 results. Only studies written in English that addressed our hypothesis that bacterial vaginosis (BV) treatment with a *Lactobacillus*-based probiotic or live biotherapeutic might reduce genital inflammation, and by extension HIV risk, were included. Although many studies investigated the ability of *Lactobacillus*-based probiotics to treat BV, none used *Lactobacillus crispatus* and only three studies included analyses of the effect on genital immune factors. None of the studies found significant differences in proinflammatory cytokines or chemokines linked to HIV acquisition between women receiving probiotic and those receiving placebo. One study showed elevated vaginal IL-5 and upregulation of genes related to proinflammatory responses and epithelial barrier function among women who received the probiotic.

### Added value of this study

Previous studies investigating the genital immune impact of BV treatment with a *Lactobacillus*-based probiotic or live biotherapeutic have only evaluated probiotics containing *Lactobacillus* species that are not typically predominant in the female genital tract and have not been linked to HIV protection in vivo. To our knowledge, this study is the first to examine the genital immune impact of a topical *L crispatus*-based live biotherapeutic (LACTIN-V) that has been shown to reduce BV recurrence compared with placebo in a large randomised controlled trial. By measuring the abundance of key vaginal bacterial taxa, we were able to explore potential mechanisms by which LACTIN-V modulates genital immunology. We showed a significant association between probiotic or live biotherapeutic use following standard BV treatment and genital immune correlates of HIV acquisition. Exploratory analyses suggest that these associations might be driven by elevated *L crispatus* abundance and reduced BV-associated anaerobe abundance.

### Implications of all the available evidence

We showed that BV treatment with a *Lactobacillus*-based live biotherapeutic might confer lower genital immune correlates of HIV risk among women. Our results differ from previous studies, although these studies did not evaluate probiotics containing *Lactobacillus* species that are common in the female genital tract, such as *L crispatus*. BV treatment with LACTIN-V following standard antibiotic treatment might represent a novel HIV prevention strategy among women.
