# Supplementary material for: Sustained effect of LACTIN-V (Lactobacillus crispatus CTV-05) on genital immunology following standard bacterial vaginosis treatment: results from a randomised, placebo-controlled trial
Source: Lancet Microbe. Author manuscript; Available in PMC 2022 Jun 11. (PMC9188188; doi:10.1016/S2666-5247(22)00043-X)
Supplement: 1 [file NIHMS1812593-supplement-1.pdf]

THE LANCET
Microbe

Supplementary appendix

This appendix formed part of the original submission and has been peer reviewed. We post it as supplied by the authors.

Supplement to: Armstrong E, Hemmerling A, Miller S, et al. Sustained effect of LACTIN-V (Lactobacillus crispatus CTV-05) on genital immunology following standard bacterial vaginosis treatment: results from a randomised, placebo-controlled trial. *Lancet Microbe* 2022; published online April 21. https://doi.org/10.1016/S2666-5247(22)00043-X.

Supplemental Appendix

Supplemental Methods

<u>Study participants</u>

Blood was collected for HIV and syphilis diagnostics and a vaginal swab was obtained for gonorrhea, chlamydia, and trichomonas diagnostics. Urine was collected to assess pregnancy status (based on human chorionic gonadotropin concentration) and to screen for urinary tract infection (based on urinalysis and bacterial culture). *Herpes simplex* virus diagnostics were not performed, but women with active genital herpes lesions were excluded. Immediately following collection, vaginal swabs were plunged into 2mL of Starplex transport medium and frozen at -20°C or -80°C. One vaginal swab was sent to the University of California San Francisco for quantification of *L. crispatus* and the *L. crispatus* CTV-05 strain, and one was sent to the University of Toronto for immune and other microbiome analyses.

<u>Quantitative polymerase chain reaction</u>

All qPCR assays performed at the University of Toronto were Taqman-based and performed on the QuantStudio 6 Flex Real-Time PCR System (Thermofisher). Protocol for quantification of *L. crispatus, L. iners, L. jensenii,* and *L. gasseri* absolute abundances in multiplex qPCR was adopted from Balashov and colleagues.[1] Absolute abundances of *G. vaginalis, A. vaginae,* and *Megasphaera* spp. were quantified in multiplex qPCR according to Kusters and colleagues.[2] Total *Prevotella* absolute abundance was quantified with qPCR adopted from Martin and colleagues.[3] Primer and probe sequences are presented in table S1. Total reaction volume for assays was 10ul. Assays for total *Prevotella*, *L. crispatus, L. iners, L. jensenii*, and *L. gasseri* were performed at 95°C for 10 min, 45 cycles at 95°C for 15 s then 60°C for 1 min. Assays for *G. vaginalis, A. vaginae,* and *Megasphaera* spp. were performed at 95°C for 10 min, 45 cycles at 95°C for 15 s then 55°C for 1 min. Lower limit of detection was determined as the Ct from no template controls. In the case of *L. gasseri*, the lower limit of detection differed between runs so the lower Ct was applied across runs. Data analysis was performed with QuantStudio Real-Time PCR Software version 1·3 (Applied Biosystems). Copy numbers were quantified using equation 1, where ΔCt represents the difference in Ct between a sample and negative control:

$$Copy\ number = 2^{\Delta Ct}$$

<u>*Lactobacillus crispatus* and CTV-05 polymerase chain reaction</u>

Polymerase chain reaction (PCR) reactions performed at the University of California San Francisco were set up in duplicate with 25ul QuantiTect SYBR Green RT-PCR Master Mix (Qiagen), 2·5uL forward primer (10uM), 2·5 uL reverse primer (10uM), 10uL extract and 10uL H2O for a total volume of 50uL. Following initial denaturation at 95°C for 15 minutes, PCR cycling consisted of 40 cycles at 95°C for 30 seconds, 58°C for 60 seconds, and 72°C for 60 seconds. Gene targets for strain and species-specific PCR were selected using genes identified in *L. crispatus* CTV-05 and *L. crispatus*-specific regions which were absent in other sequenced bacterial strains and with low numbers of homologs in vaginal metagenome datasets. Primer sequences are presented in table S2. Bacterial concentration was calculated from mean sample Ct values using a standard curve based on serial dilutions of CTV-05 strain of *L. crispatus*. Limits of detection determined for each target at the 95% detection threshold were 6·0 x 10E2 CFU/mL (CTV-05 strain) and 9·53 x 10E2 CFU/mL (*L. crispatus*). Detection of *L. crispatus* and *L. crispatus* CTV-05 were defined as above the lower limit of detection as defined above.

<u>Statistics</u>

To evaluate the mediating effect of key bacterial taxa on the relationship between LACTIN-V treatment and levels of soluble immune factors at 24 weeks, we followed the framework established by Baron and Kenny.[4] Soluble immune factors and bacterial taxa that were significantly associated with treatment were included in mediation analyses. First, we established whether there was an association between the potential mediator (bacterial abundance) and outcome (soluble immune factor levels) with linear regression. Change in soluble immune factors and bacterial abundance from baseline to 24 weeks were incorporated into these (and subsequent) models to allow us to control for baseline variation in both variables simultaneously. Next, linear regression models were generated that included change in a soluble immune factor from baseline to 24 weeks as the dependent variable and treatment group and change in abundance of one of the bacterial taxa from baseline to 24 weeks as independent variables.

Supplemental figure S1. *L. crispatus* and *L. crispatus* strain CTV-05 among participants receiving LACTIN-V or placebo. Proportion of individuals who received LACTIN-V with detectable *L. crispatus* and *L. crispatus* strain CTV-05 based on treatment allocation. P values determined with Pearson Chi-Square test.

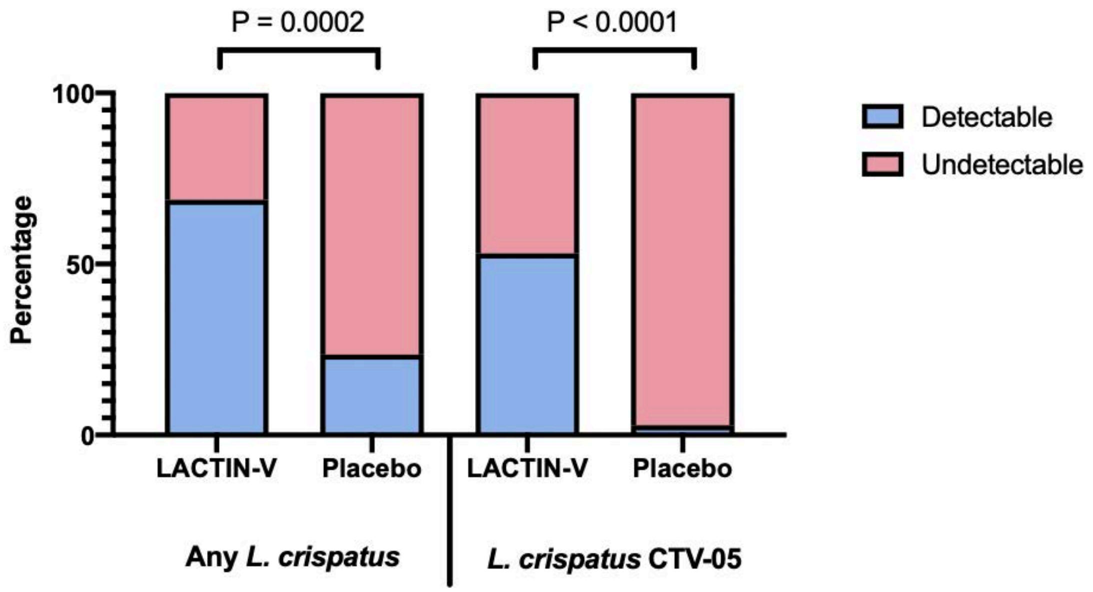

Supplemental figure S2. Predominance by CTV-05 and non-CTV-05 *L. crispatus* among participants with high *L. crispatus*. Proportion of individuals with predominance by *L. crispatus* CTV-05 and non-CTV-05 strains among those with >1x10^6 copies per mL of *L. crispatus*.

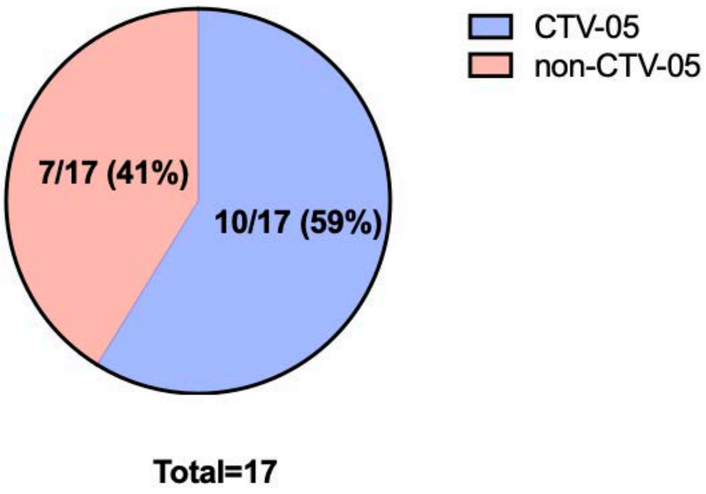

Supplementary table S1. Primer and probe sequences for quantitative polymerase chain reaction assays.

Target	Oligo	Sequence
L. crispatus	Forward	CGTGGTTCAGCWTTGAAGGC
	Reverse	CTTCAACTGGCATYAAGAATGGC
	Probe	[ROX]-AGGCGACAAGGAAGCTCAAGAAC-BHQ2
L. iners	Forward	CGTGGTTCAGCWTTGAAGGC
	Reverse	CTTCAACTGGCATYAAGAATGGC
	Probe	[HEX]-AGGCGATCCAGAACAAGAAGCAG-BHQ1
L. gasseri	Forward	CGTGGTTCAGCWTTGAAGGC
	Reverse	CTTCAACTGGCATYAAGAATGGC
	Probe	[FAM]-AGGTGACCCAGAACAACAAGACG-BHQ1
L. jensenii	Forward	CGTGGTTCAGCWTTGAAGGC
	Reverse	CTTCAACTGGCATYAAGAATGGC
	Probe	[Cy5]-AGGTGACCCAGAACAAGAAAAGGT-BHQ2
G. vaginalis	Forward	GCGGGCTAGAGTGCA
	Reverse	ACCCGTGGAATGGGCC
	Probe	[ROX]CTTCTCAGCGTCAGTAACAGC
A. vaginae	Forward	TAGGTCAGGAGTTAAATCTG
	Reverse	TCATGGCCCAGAAGACCGCC
	Probe	[HEX]CTACCAGACTCAAGCCTGCC
Megasphaera	Forward	GATGCCAACAGTATCCGTCCG
	Reverse	CCTCTCCGACACTCAAGTTCGA
	Probe	[FAM]ACAGACTTACCGAACCGCCT
Prevotella	Forward	5-CCAGCCAAGTAGCGTGCA-3
	Reverse	5-TGGACCTTCCGTATTACCGC-3
	Probe	(56-FAM)-AATAAGGACCGGCTAATTCCGTGCCAG-(36-TAMSp)

Supplementary table S2. Primer sequences for *L. crispatus* and CTV-05 polymerase chain reaction assays.

Target	Oligo	Sequence
L. crispatus	Forward	AAAGTCCTGGTTTGATCTGCGT
	Reverse	CACTTCCTAGCCACTGTGTTGT
L. crispatus CTV-05	Forward	GCTGTTGCAGCCAGACAGTT
	Reverse	TCTCTGGGACATCCATAAGTTG

Supplementary table S3. Association between treatment and detectability of soluble immune factors.

	Independent variable: Treatment group		
**Dependent variables**	**B coefficient**	**Std. error**	**P value**
IFN-α2a	-0.405	0.521	0.436
IL-17A	0.121	0.569	0.831
Baseline measurements of immune factor detectability included in each model to control for inter-individual variation. Treatment group recoded such that LACTIN-V group = 1 and placebo group = 2. Shading of rows represents discrete linear regression models.			

References

1. Balashov S V., Mordechai E, Adelson ME, Sobel JD, Gygax SE. Multiplex quantitative polymerase chain reaction assay for the identification and quantitation of major vaginal lactobacilli. *Diagn Microbiol Infect Dis*. 2014;78:321-327. doi:10.1016/j.diagmicrobio.2013.08.004
2. Kusters JG, Reuland EA, Bouter S, Koenig P, Dorigo-Zetsma JW. A multiplex real-time PCR assay for routine diagnosis of bacterial vaginosis. *Eur J Clin Microbiol Infect Dis*. 2015;34:1779-1785. doi:10.1007/s10096-015-2412-z
3. Martin FE, Nadkarni MA, Jacques NA, Hunter N. Quantitative microbiological study of human carious dentine by culture and real-time PCR: Association of anaerobes with histopathological changes in chronic pulpitis. *J Clin Microbiol*. 2002;40:1698-1704. doi:10.1128/JCM.40.5.1698-1704.2002
4. Baron RM, Kenny DA. The moderator-mediator variable distinction in social psychological research: Conceptual, strategic, and statistical considerations. *J Pers Soc Psychol*. 1986. doi:10.1037//0022-3514.51.6.1173
